# Older Workers and Affective Job Satisfaction: Gender Invariance in Spain

**DOI:** 10.3389/fpsyg.2018.00930

**Published:** 2018-06-08

**Authors:** Juan J. Fernández-Muñoz, Gabriela Topa

**Affiliations:** ^1^Department of Medicine and Surgery, Psychology, Preventive Medicine and Public Health, Immunology and Medical Microbiology, Nursing, and Stomatology, Universidad Rey Juan Carlos, Madrid, Spain; ^2^Department of Social and Organizational Psychology, Universidad Nacional de Educación a Distancia (UNED), Madrid, Spain

**Keywords:** affective satisfaction, multi-group analysis, job involvement, gender, invariance testing

## Abstract

Older employees’ affective job satisfaction is an aspect that arouses growing interest among researchers. Among the affective measures of job satisfaction, the Brief Index of Affective Job Satisfaction (BIAJS) is one of the most used in the last decade. This study is intended to the test the gender invariance of the BIAJS in two samples of workers over age 40 in Spain. The first sample, of 300 participants and the second sample, of 399 participants, have been used to test gender invariance of the BIAJS. In comparison with the original English version, the Spanish version of the BIAJS has adequate psychometric properties. The findings allow us to consider it a valid and reliable tool to assess older people’s affective expressions about their work. In addition, this study provides evidence of its factorial invariance as a function of gender.

## Introduction

Older employees’ affective job satisfaction is an aspect that arouses growing interest among researchers (Miao et al., 2016, 2017; Davies et al., 2017). In general, the influence of the affective facets (or aspects, dimensions) of work on other outcomes, such as the employee’s individual performance and the achievement of organizational goals, is acknowledged (Saber, 2014; Cantarelli et al., 2016). As a result, interest in the evaluation of affective job satisfaction is as up-to-date nowadays as it was more than 60 years ago (Brayfield and Rothe, 1951), when the first emotional indicators of job satisfaction were proposed. However, since women’s presence in the workplace is now very common, the affective evaluation of job satisfaction must be carried out with instruments whose invariance across genders cannot not be questioned (Collins et al., 2014; Karin Andreassi et al., 2014). Finally, population aging and the prolongation of working life make the evaluation of older workers’ job satisfaction a highly topical theme (Lytle et al., 2015).

Among the affective measures of job satisfaction, the Brief Index of Affective Job Satisfaction (BIAJS) is one of the most used in the last decade (Hurtado et al., 2017; Kottwitz et al., 2017). Previous studies adequately documented its temporal stability, and cross-national and cross-population invariance, by job level and work organizations, but not its invariance as a function of gender (Thompson and Phua, 2012). Given that the literature documents the existence of gender-specific patterns of emotions, the evidence about gender invariance of job-related affective measures is a topic of interest (Judge et al., 2017). As a result, this database allows us to the test the gender invariance of the BIAJS in two samples of workers over age 40 in Spain.

### Affective Measures vs. Cognitive Measures of Job Satisfaction

Affective job satisfaction is a global and positive emotional response to one’s work as a whole (Moorman, 1993). Affective job satisfaction is often considered synonymous with general or overall satisfaction and it is evaluated through items that ask people how much they like their work. In contrast, the evaluation of the cognitive facets of job satisfaction arises from the rational comparison of work conditions with a desired, expected, or promised standard (Locke, 1969; Moorman, 1993; Spector, 1997). Accordingly, it seems clear that emotional job satisfaction is a different construct, although partially related to cognitive satisfaction.

Whereas job satisfaction is the most researched construct in the psychology of work and organizations, there is still no consensus about its structure (Judge et al., 2017). The conceptual and statistical relations between cognitive and affective job satisfaction are a source of controversy (Thompson and Phua, 2012). Whereas some argue that emotional satisfaction directly reflects all the cognitive facets of satisfaction, others insist that it only covers some specific aspects of the cognitive measures (Judge and Ilies, 2004). In this sense, it would still be problematic to establish which facets should be included and what relative weight they would have in the overall assessment (Thompson and Phua, 2012).

Many authors criticize that job satisfaction is generally defined as emotional, but it is evaluated in its cognitive aspects (Moorman, 1993). In this sense, the development of specifically affective measures of job satisfaction, such as the BIAJS, has received a major boost in the last decade (Pellegrini et al., 2010), as has the search for evidence on the invariance of this instrument (Thompson and Phua, 2012). However, cross-population equivalence tests have been based on nationality, job level, and job type, but not on gender. On another hand, the studies have relied almost exclusively on English-speaking samples (Pu et al., 2017), whereas studies related to affective job satisfaction with older Spanish-speaking employees are much rarer (Topa and Alcover, 2015). Lastly, although the initial drafting of the BIAJS items was based on interviews of older workers, with an age mode between 45 and 49 years, gender invariance with older workers was not tested.

So, the current data set includes the assessment of the BIAJS in a first sample of workers over 40, and in a second sample of workers over 44. It contributes to existing research by providing data from (a) two different samples of older workers, (b) correlations with other theoretically related variables (job involvement), and (c) it provides sociodemographic data. It therefore enables researchers to further explore the associations between BIAJS and older workers’ attitudes and behaviors, as well as potential moderators of these associations (education, job type, etc.).

## Method

### Ethics Statement

The Ethical Committee of the third author’s University Bio-ethical Committee of the National Distance Education University (UNED) approved the Project in May 2014. The ethical standards for research of the Declaration of Helsinki revised in Fortaleza (World Medical Association, 2013) have been followed.

### Participants

#### Sample 1

The first sample, of 300 participants, was split into two groups: males (164) and females (136). The males’ group was composed of 164 participants with an average age of 53.01 years (*SD* = 4.94) and an age range of 44–63; educational attainment: 7.3% had completed basic education; 33.5% had completed secondary level studies, and 59.1% had finished university studies; regarding the females’ group, the average age was 53.27 years (*SD* = 4.93) with an age range of 45–65; educational attainment: 5.1% had completed basic education; 32.4% had completed secondary level studies, and 62.5% had finished university studies.

#### Sample 2

The second sample, of 399 participants, was also split into two groups: males (243) and females (156); regarding the males’ group, the average age was 61.51 years (*SD* = 2.42) with an age range of 45–66; educational attainment: 34.2% had completed basic education; 30.9% had completed secondary level studies, and 35% had finished university studies. The females’ group was composed of 156 participants with an average age of 60.84 years (*SD* = 3.18) and an age range of 45–66; educational attainment: 32.3% had completed basic education, 28.3% had completed secondary level studies, and 39.3% had finished university studies.

### Procedure

We developed the Spanish version of the BIAJS scale in two steps. First, the original seven-item BIAJS in English (Thompson and Phua, 2012) was translated to the Spanish context. Various experts in work and organizational psychology drafted the items based on the original version in English. Next, back-translation was carried out by a native English speaker, who was unaware of the contents of the original scale. We then compared the outcome with the original version of the questionnaire. Secondly, we administered the BIAJS scale to the two samples that make up the present study (Sample 1: workers between 44 and 65 and Sample 2: workers between 44 and 66). This step of the study was carried out by means of questionnaires distributed in different organizations by the collaborators of the research team, who performed the task after having received precise instructions to homogenize the administration procedures of the tests. The participants were informed of the goals of the study, of the anonymity of the data collected, and they expressed their consent, after which they completed the workbook containing the diverse scales of the study.

#### Instruments

The BIAJS (Thompson and Phua, 2012). This brief scale is a measurement instrument of Affective Job Satisfaction with just one factor composed of four items: “I find real enjoyment in my job,” “I like my job better than the average person,” “Most days, I am enthusiastic about my job” and “I feel fairly well satisfied with my job.” Responses are rated on a five-point Likert scale ranging from 1 = strongly disagree to 5 = strongly agree. Moreover, to reduce priming effects and acquiescent answers, the scale includes three distracter items: “My job is unusual” (between Item 1 and 2), “My job needs me to be fit” (between item 2 and 3, and “My job is time consuming” (between Item 3 and 4) (see **Annex I**).

Regarding the psychometric properties, alpha coefficient was 0.83, and corrected item-total correlations were between 0.64 and 0.70. The fit indices for the confirmatory factor analysis were: GFI (*goodness of fit index*) = 0.95, CFI (*comparative fit index*) = 0.93, NFI (*normed fit index*) = 0.93, RMR (*root mean square residual*) = 0.05, and RMSEA (*Root Mean Square Error of Approximation*) = 0.06; the test-retest score for temporal stability was 0.57 (*p* < 0.01), and the concurrent validity, tested with the Satisfaction with Life Scale (SWLS) (Diener et al., 1985) was *r* = 0.51 (*p* < 0.01).

Job Involvement Scale (Kanungo, 1982): The Job Involvement Questionnaire was used, including four items. Examples of items are “Most of my interests are focused on my job,” “I consider my job to be very central to my existence.” Response scale ranged from 1 (*Totally disagree*) to 5 (*Totally agree*). This scale reached α = 0.83 in a previous study (Topa and Alcover, 2015).

## Preliminary Statistical Analysis

We performed a preliminary factor analysis on the BIAJS (results reported in **Table 1**) to better explain their relevance. The statistical analyses were performed with SPSS 22 and AMOS 19 (Byrne, 2016). Previous assumptions were tested to ensure the applicability of exploratory factor analysis (EFA) and confirmatory factor analysis (CFA): large sample size, Kaiser-Meyer-Olkin (KMO) index and the Bartlett test of sphericity, multivariate normality, linearity, and correlation between variables (Tabachnick and Fidell, 2013).

**Table 1:** Means, standard deviations, item homogeneity, α if item is deleted, and inter-item correlation for the four items of the Brief Index of Affective Job Satisfaction (BIAJS) between Males and Females.

			*M*	*SD*	Item homogeneity	1	2	3
***N* = 300**	Males	BIAJS_1	3.35	1.10	0.771			
		BIAJS_2	3.17	1.17	0.710	0.628^∗^		
		BIAJS_3	2.91	1.10	0.757	0.699^∗^	0.637^∗^	
		BIAJS_4	3.36	1.10	0.752	0.693^∗^	0.626^∗^	0.652^∗^
	Females	BIAJS_1	3.37	1.06	0.699			
		BIAJS_2	3.16	1.09	0.608	0.474^∗^		
		BIAJS_3	3.03	0.981	0.711	0.606^∗^	0.588^∗^	
		BIAJS_4	3.50	0.999	0.726	0.703^∗^	0.522^∗^	0.605^∗^
***N* = 399**	Males	BIAJS_1	3.36	1.01	0.801			
		BIAJS_2	3.03	1.10	0.722	0.683^∗^		
		BIAJS_3	3.10	0.978	0.744	0.732^∗^	0.613^∗^	
		BIAJS_4	3.45	0.923	0.706	0.653^∗^	0.612^∗^	0.609^∗^
	Females	BIAJS_1	3.37	1.12	0.729			
		BIAJS_2	3.07	1.18	0.648	0.687^∗^		
		BIAJS_3	3.11	0.981	0.627	0.503^∗^	0.417^∗^	
		BIAJS_4	3.37	1.11	0.757	0.633^∗^	0.558^∗^	0.718^∗^


*^∗^All correlations were significant at *p* < 0.01.*

For the Confirmatory Factor Analysis, the adequacy of the model was tested within the absolute fit indices with weighted least squares and with adjusted mean and variance criteria. It is recommended to estimate thresholds when equal or fewer than five response categories are used (Beauducel and Herzberg, 2006). For instance: the chi-square statistic (χ^2^) (Jöreskog and Sörbom, 1979); the adjusted the goodness of fit index (AGFI), whose acceptable value reference is over 90 (Hu and Bentler, 1999); the CFI, the NFI and the Tucker Lewis index (TLI) -in all three cases, the values range between 0 and 1, and the reference value is 0.90 (Bollen, 1989; Bentler, 1990) – and, within parsimony fit indices, the RMSEA and the RMR, the smaller their value, the better the fit, the reference value being 0.05 (Steiger and Lind, 1980; Cheung and Rensvold, 2002). All indices were calculated for the male and female groups and the total sample. For the multigroup analysis between male and females the fit indices used to test the factorial invariance were applied according to Vandenberg and Lance (2000).

Sample 1: the BIAJS scale showed an internal consistency for males of 0.884, and for females, of 0.847 (Cronbach’s alpha). Regarding the males, item homogeneity was between 0.752 and 0.771 and, for females; it was a bit lower, between 0.608 and 0.726.

Sample 2: Cronbach’s alpha was 0.880 for males, and 0.849 for females, and item homogeneity was between 0.706–0.801 and 0.627–0.757, respectively, for males and females. **Table 1** presents descriptive analysis with means, standard deviations and correlations for each item of the scale and for both samples.

For Sample 1 and for the male group, Bartlett’s sphericity test obtained χ^2^ = 343.228 (*df* = 6), *p* < 0.001, the KMO value was 0.838; on the other hand, for the female group, Bartlett’s sphericity test obtained χ^2^ = 229.503 (*df* = 6), *p* < 0.001, and the KMO value was 0.789. The factorial weights for males were between 0.878 and 0.835 and for females between 0.770 and 0.858.

For Sample 2, the data findings were similar to the sample 1; for males: KMO = 0.828, and Bartlett’s test χ^2^ = 513.087 (*df* = 6), *p* < 0.001, and, for females, KMO = 0.741, and Bartlett’s test χ^2^ = 295.664 (*df* = 6), *p* < 0.001. The factorial weights for males were between 0.694–0.804 (73.82% explained variance) and, for females, it was between 0.25–0.775 (69.08% explained variance). For both samples, the EFA has showed one unidimensional factorial solution.

Confirmatory Factor Analysis was carried out on the data by sample and group to replicate the single factor solution of the BIAJS. Thus, the four items of the scales were expected to load on a single factor (see **Table 2**).

**Table 2:** Fit indices for the BIAJS from Confirmatory Factor Analysis for both samples across gender variable.

		χ^2^/*df*	*df*	*p*	AGFI	CFI	NFI	TLI	RMR	RMSEA
***N* = 300**	Males	0.473	2	0.623	0.986	0.998	0.997	0.992	0.010	0.00
	Females	0.787	2	0.013	0.971	0.972	0.964	0.917	0.011	0.00
	Total	1.24	2	0.033	0.979	0.992	0.989	0.976	0.009	0.02
***N* = 399**	Males	1.28	2	0.352	0.973	0.999	0.995	0.997	0.012	0.03
	Females	0.420	2	0.011	0.986	0.925	0.923	0.658	0.007	0.00
	Total	0.421	2	0.038	0.899	0.991	0.989	0.944	0.020	0.00


**AGFI, adjusted goodness of fit index; CFI, comparative fit index; NFI, normed fit index; Tucker-Lewis coefficient; RMR, root mean square residual; RMSEA, root mean square error of approximation*.*

On the one hand, for the sample *n* = 300 the findings for the multigroup analysis, comparison between several models were: measurement weights, NPAR (Number of distinct Parameters): 13, χ^2^: 21.40, *df*: 7, *p*: 0.003, χ^2^/*df*: 3.057, and RMSEA: 0.072; structural covariances, NPAR: 12, χ^2^: 21.40, *df*: 8, *p*: 0.006, χ^2^/*df*: 2.67 and RMSEA: 0.065; measurement residuals, NPAR: 8, χ^2^: 30.77, *df*: 12, *p*: 0.002, χ^2^/*df*: 2.56, and RMSEA: 0.063; and independence model: NPAR: 8, χ^2^: 96.36, *df*: 12, *p*: 0.000, χ^2^/*df*: 8.03, and RMSEA: 0.13. On the other hand for the sample = 399 the results were: measurement weights, NPAR: 13, χ^2^: 10.39, *df*: 7, *p*: 0.171, χ^2^/*df*: 1.47, and RMSEA: 0.040; structural covariances, NPAR: 12, χ^2^: 12.38, *df*: 8, *p*: 0.135, χ^2^/*df*: 1.54, and RMSEA: 0.043; measurement residuals, NPAR: 8, χ^2^: 14.37, *df*: 12, *p*: 0.277, χ^2^/*df*: 1.19, and RMSEA: 0.026; and independence model: NPAR: 8, χ^2^: 58.14, *df*: 12, *p*: 0.000, χ^2^/*df*: 48.34, and RMSEA: 0.39.

Finally, to test the criterion validity, the BIAJS was correlated with the other theoretical latent variables (constructs) related to affective job satisfaction. Significant correlations were found between affective job satisfaction and the Construct Job Involvement Scale, *r* = 0.262, *p* < 0.01. For each group, the correlations were also significant: *r* = 0.276, *p* < 0.01, for males, and *r* = 0.242, *p* < 0.01, for females.

For Sample 2, the correlations were also significant although weaker than in Sample 1. For all the entire sample, the correlation was *r* = 0.189, *p* < 0.01; and for the males, it was *r* = 0.179, *p* < 0.01, and for the females, *r* = 0.205, *p* < 0.05.

## Suggestions of Future Avenues of Research Using This Data Set

This data set would be tied in with research into the influence of affective job satisfaction on workers’ desirable attitudes and behaviors. Meta-analytical reviews and individual studies frequently explore differences as a function of sociodemographic characteristics, such as race (Koh et al., 2016), or employment conditions, multiple versus single job holders (Kottwitz et al., 2017), or hierarchical level (Hurtado et al., 2017). However, studies that analyze factor structure invariance as a function of gender are more uncommon in job-related affective well-being measures, such as that of Laguna et al. (2017). To our knowledge, there is no study that has examined this psychometric property in relation to the BIAJS in older Spanish workers. As the literature shows that job satisfaction is a relevant predictor associated with the planning of and decision to retire or to continue working (Topa et al., 2018), this analysis seems relevant.

In comparison with the original English version, the Spanish version of the BIAJS has adequate psychometric properties. The findings allow us to consider it a valid and reliable tool to assess older people’s affective expressions about their work. In addition, this set of data provides evidence of its factorial invariance as a function of gender.

In this sense, according to the internal consistency of the scale, this data set has shown similar reliability for both samples and groups as the original instrument (Thompson and Phua, 2012); likewise, homogeneity indices were higher in general than those of Thompson and Phua (2012). Regarding the fit indices, all were excellent for both samples and groups, showing factorial stability across gender for this scale. Also, the concurrent validity with the Job Involvement Scale (Kanungo, 1982) was tested for both samples and groups, with adequate findings. Some significant correlations were a bit weak, specifically, for Sample 2 and for the females.

Concerning the size and representativeness of the samples, the limitations of this data are obvious, especially those due to the sampling procedure used. Moreover, all the data proceed from self-reports, which can include a source of uncontrolled error from the common variance. However, because the BIAJS is focused on subjective evaluations of one’s job, deviations from external criteria would not necessarily indicate that the BIAJS is an invalid instrument. In summary, we conclude that the available dataset could be used to expand research on job satisfaction and aging among Spanish-speaking populations (Unanue et al., 2017), and empirically support further theoretical development. In this regard, future analyses could reuse the data to develop practical interventions for subgroups of older workers who show weaker job satisfaction in order to improve their desirable job attitudes.

## Data Set Description

The data set, called *Aged Worker’s Job Satisfaction*, can be found in Figshare repository and is accessible through the following hyperlink: https://figshare.com/articles/BIAJS_Brief_Index_of_ Affective_Job_Satisfaction/5806743.

The file named Sample 1 (Sample1_300.sav) contains the individual responses of the 300 participants on the seven items of the BIAJS scale, and job involvement assessment along with demographic data (age, gender, education, type of employment contract, and organizational seniority). The file named Sample 2 contains the 399 individual responses (Sample2_399.sav) on the seven items of the BIAJS scale, and job involvement assessment along with demographic data (age, gender, education, type of employment contract, and organizational seniority).

## Author Contributions

All authors listed have made a substantial, direct and intellectual contribution to the work, and approved it for publication.

## Conflict of Interest Statement

The authors declare that the research was conducted in the absence of any commercial or financial relationships that could be construed as a potential conflict of interest.

## Annex I

The Brief Index of Affective Job Satisfaction (BIAJS).

I find real enjoyment in my job

My job is unusual (distracter)

I like my job better than the average person

My job needs me to be fit (distracter)

Most days I am enthusiastic about my job

My job is time consuming (distracter)

I feel fairly well satisfied with my job

*Interval measure:* 1 = *Strongly disagree*, 2 = *Disagree*, 3 = *Neutral*, 4 = *Agree*, 5 = *Strongly agree.*

The Brief Index of Affective Job Satisfaction (BIAJS)-Spanish version.

Disfruto realmente de mi trabajo

Mi trabajo es especial (distracter)

Me gusta mi trabajo más que a la mayoría de las personas

Mi trabajo me exige estar en forma (distracter)

La mayor parte de los días estoy entusiasmado en mi trabajo

Mi trabajo me consume mucho tiempo (distracter)

Me siento realmente muy satisfecho con mi trabajo.

*Scale response:* 1 = *totalmente en desacuerdo*, 2 = *en desacuerdo*, 3 = *ni de acuerdo ni en desacuerdo*, 4 = *de acuerdo*, 5 = *totalmente de acuerdo.*
